# Spontaneous abortion and ectopic pregnancy: Case definition & guidelines for data collection, analysis, and presentation of maternal immunization safety data

**DOI:** 10.1016/j.vaccine.2017.01.047

**Published:** 2017-12-04

**Authors:** Caroline E. Rouse, Linda O. Eckert, Isaac Babarinsa, Emily Fay, Manish Gupta, Margo S. Harrison, Alison Tse Kawai, Elyse O. Kharbanda, Merita Kucuku, Lee Meller, Tamala Mallett Moore, Maja Subelj, Sonali Kochhar, Fernanda Tavares-Da-Silva, for the Global Alignment of Immunization Safety in Pregnancy (GAIA) Abortion Work Group, The Brighton Collaboration Abortion Working Group

**Affiliations:** a Brigham and Women’s Hospital, Boston, MA, USA; b University of Washington, Seattle, WA, USA; c Sidra Medical and Research Center/Weill Cornell Medicine-Qatar/Women’s Hospital, Doha, Qatar; d Barts Health NHS Trust, London, UK; e Columbia University Medical Center, New York, NY, USA; f Harvard Medical School and Harvard Pilgrim Health Care Institute, Boston, MA, USA; g HealthPartners Institute, Minneapolis, MN, USA; h Department of Vaccines Control, National Agency for Medicines & Medical Devices, Albania; i Sanofi Pasteur, Swiftwater, PA, USA; j National Institute of Public Health, Ljubljana, Slovenia; k Global Healthcare Consulting, India; l GlaxoSmithKline Biologicals, Wavre, Belgium; m Erasmus University Medical Center, Rotterdam, The Netherlands

**Keywords:** Abortion, Miscarriage, Ectopic, Adverse event, Immunization, Guidelines, Case definition

## 1 Preamble

### 1.1 Need for developing case definitions and guidelines for data collection, analysis, and presentation for spontaneous abortion and ectopic pregnancy as adverse events following immunization during pregnancy

Vaccine-preventable infectious diseases are responsible for maternal, morbidity and mortality. Immunization of pregnant women can protect against vaccine-preventable infections, and may have the added benefit of direct fetal protection. Outcomes of spontaneous abortion and ectopic pregnancy following maternal receipt of vaccination have been less studied. There have been few prospective clinical trials evaluating vaccination in pregnancy; most safety data available are derived from registries where outcomes are passively reported.

Spontaneous abortion and ectopic pregnancy are important pregnancy outcomes that should be included in vaccine registries or included as important outcomes in vaccine research. As many organizations define pregnancy loss uniquely we will compare and contrast the existing definitions and provide guidance for use of this adverse event term in studies of maternal immunization.

**Table:** 

Abbreviations used	
β-HCG	beta human chorionic gonadotropin
HPV	human papillomavirus
IIV	Inactivated influenza vaccines
MMR	Measles, mumps, rubella
MR	Measles, rubella
SA	Spontaneous abortion
TT	Tetanus toxoid
Td	Tetanus, diphtheria vaccine
TdaP	Tetanus, diphtheria, pertussis vaccine
TVUS	Transvaginal ultrasound
VAERS	Vaccine Adverse Event Reporting System
WHO	World Health Organization

*Definition and diagnosis of spontaneous abortion and ectopic pregnancy*

*First trimester spontaneous abortion (Less than 14 weeks 0 days gestation)*

Many terms have been used to describe the failure of an early pregnancy, including: early pregnancy loss, early pregnancy failure, miscarriage, and spontaneous abortion. Pregnancy failure can be further classified as inevitable, missed, anembryonic, or embryonic demise [1], [2]. For the purposes of this document, we will use the term “spontaneous abortion” (“SA”) to describe early pregnancy loss. Various national and international organizations have released guidelines for the diagnosis and/or workup of suspected early or first trimester spontaneous abortion, which are presented in the Tables.

*Second trimester spontaneous abortion (Between 14 weeks 0 days and 21 weeks 6 days)*

The arbitrary division by gestational age between abortion and stillbirth complicates the definition and diagnostic criteria for second trimester abortion. Existing definitions are outlined in the Tables.

*Ectopic pregnancy*

Ectopic pregnancy is one in which the pregnancy implants in a location other than the uterine endometrium. While most ectopic pregnancies occur in the fallopian tube (up to 97%), pregnancies can also implant in the abdomen, cervix, ovary and cornua of the uterus [3]. Society guidelines agree that the evaluation of a woman with a pregnancy of uncertain location should include an ultrasound examination followed by serum measurement of beta human chorionic gonadotropin (β-HCG) level if no intrauterine pregnancy is identified by ultrasound. If the serum β-HCG is above the discriminatory zone (the serum β-HCG level at which an intrauterine pregnancy should be visible, generally around 1500–2000 mIU/ml) and no intrauterine pregnancy is identified, an ectopic pregnancy is likely [3], [4], [5]. It should be noted that these society guidelines are primarily applicable for high resource settings given reliance on ultrasound for diagnosis, whereas the definitions in this document can be applied to all settings.

*Induced abortion*

While a full case definition for induced abortion is not included in this document, we recommend reporting this as a pregnancy outcome of interest. Induced abortion is the termination of pregnancy through medical or surgical procedures. Guidelines for safe, comprehensive care of women with induced abortion have been published by many groups, including the World Health Organization (WHO), the American Congress of Obstetricians and Gynaecologists (ACOG), the Royal College of Obstetricians and Gynaecologists (RCOG), the Royal Australian and New Zealand College of Obstetricians and Gynaecologists (RANZCOG), and the Society of Obstetricians and Gynaecologists of Canada (SOGC) [6], [7], [8], [9], [10], [11].

*Epidemiology of spontaneous abortion and ectopic pregnancy*

Spontaneous abortion (SA) is a common outcome. Published frequency of SA reported by several authors varies depending on the definition used [12], [13], [14], [15], [16], [17], [18], [19], [20], [21], [22], [23], [24], [25], [26], [27]. In one systematic review study, the cumulative risk of SA for weeks 5 through 20 of gestation ranged from 11 to 22 miscarriages per 100 women (11–22%) [28]. This number varies by age group and study population, with women over 35 years of age experiencing the highest rates of SA [29], depending on gestational age, with a higher risk of SA earlier in gestation [30].

Ectopic pregnancy is a serious adverse pregnancy outcome and is one of the most common causes of maternal mortality in early pregnancy [31]. Because, particularly in high resource areas, it can be treated in the outpatient setting (the visits for which are not easily tracked) confirming the incidence of ectopic pregnancy is challenging. Reported rates range generally range from 0.6% to 2.4% [32], [33], [34], [35], [36]. These rates may be increasing secondary to an increase in the use of assisted reproductive technologies and in pelvic infection [36]. The case mortality rate varies between low and high resource settings. The mortality rate for ectopic pregnancy in the United Kingdom is 3.6/10,000 cases [37]; this rate is ten times higher in developing countries [38], which may be in part explained by the difficulty diagnosing and managing ectopics in regions with limited availability of ultrasound and/or quantitative HCG.

*Causes and risk factors of spontaneous abortion*

The most common and well-documented cause of spontaneous abortion is aneuploidy, or abnormal chromosome number (genetic factors) [39]. Studies have shown that approximately 50% of spontaneous abortions are associated with fetal chromosomal abnormalities [39]. Many studies have shown that maternal age is also a risk factor for SA. A Danish registry study that examined the outcomes of more than 1.2 million pregnancies [29] demonstrated that the risk of spontaneous loss is three times higher in women age 40 or older as compared to the under 25 age group, making age a stronger risk factor than any other known effect [39]. Other risk factors include paternal age, previous pregnancy loss, thyroid abnormalities, pre-gestational diabetes, congenital uterine anomalies, exposure to lead, mercury, organic solvents and ionizing radiation, smoking and alcohol use [39]. A recent UK population based case-control study, found the following factors to be independently associated with SA after adjustment for confounding: high maternal age, previous SA, previous pregnancy termination and infertility, assisted conception, low pre-pregnancy body mass index, regular or high alcohol consumption, feeling stressed (including trend with number of stressful or traumatic events), high paternal age and conception occurring after a change in partner [40]. Additionally, multiparity carries a risk of reproductive failure, so pregnancy order, desired family size, and maternal age should be used in consideration of the etiology of SA [41]. Paternal exposures should also be considered when studying SA because theoretically any exposure of either parent prior to conception (i.e. males during spermatogenesis and females around time of conception and during pregnancy) could increase the risk of spontaneous loss [41]. Importantly, the risk of spontaneous abortion is inversely related to week of gestation; in one study, for example, the risk of SA at 6 completed weeks of gestation was found to be 9.4% while the risk at 10 completed weeks was 0.7% [42].

Several studies have suggested that certain psychological factors can contribute to the risk for SA, such as affective disorders (depression, dysthymia and mania), and anxiety disorders, including: agoraphobia, generalized anxiety disorder, panic disorder, simple phobia, social phobia and posttraumatic stress disorder [40], [43], [44], [45], [46], [47], [48], [49], [50], [51], [52], [53], [54].

*Causes and risk factors of ectopic pregnancy*

Prior tubal surgery, in particular tubal ligation, is associated with very high rates of ectopic pregnancy. A large retrospective cohort study showed that while sterilization failure after tubal ligation is rare (0.1 to 0.8% in the first year after the procedure), approximately 1/3 of the resulting pregnancies were ectopic [55]. Use of an intrauterine device (IUD) is similarly associated with very low rates of pregnancy (0.5 per 100 users in 5 years for the levonorgesterel [LNG] device), but high rates of ectopic pregnancy (1 in 2) for those women who did conceive with the LNG-IUD in situ [56]. For women who conceive with the copper containing IUD in site, the ectopic pregnancy rate is 1 in 16 [57].

A prior history of ectopic pregnancy is another important risk factor for ectopic pregnancy, with recurrence rates ranging from 8 to 15%, depending on the modality used to treat the previous ectopic [58]. Women with a history of diethylstilbestrol exposure in utero also have an increased risk of ectopic pregnancy, with rates 9 times higher than baseline [59]. Pelvic infection, including that resulting from gonorrhea or chlamydia, is a major contributor to ectopic pregnancy risk. The rate of ectopic pregnancy in a woman with a history of one chlamydial infection was found to be 0.13%; this rate increased to 0.49% after two chlamydial infections, and rose to 1.4% after three or more infections [60]. Multiple reports have found an increase in ectopic pregnancy risk with assisted reproductive technology, with rates ranging from 2.2% to 4.5% [61], [62], [63].

*Spontaneous abortion following immunization*

Data from clinical trials and observational studies support the safety of inactivated vaccines or toxoids (e.g., tetanus, pertussis or influenza) for maternal immunization in many parts of the world.

a. Influenza vaccines:

Inactivated influenza vaccines (IIV) are recommended for use in pregnant women regardless of trimester due to the increased risk of infection and complications during pregnancy [64], [65], [66]. Systematic reviews for inactivated influenza virus vaccines did not find an association with SA and IIV, although the majority of maternal immunization studies are focused on the vaccines containing the influenza A (H1N1) pandemic antigen and limited data exists during the first trimester [67], [68], [69], [70]. Preliminary results of 2010–11 and 2011–12 seasons’ data show an increased risk of SA following IIV among pregnant women in the 1–28 day risk window who had received a pH1N1-containing vaccine the prior influenza season, adjusted odds ratio (aOR) 2.0 (95% CI 1.1–3.6) [71]. However these findings are inconsistent with prior research on IIV safety in pregnancy. Safety studies continue, and follow-up studies are planned in more recent influenza seasons.

b. Tetanus-containing vaccines:

Fewer data exist regarding spontaneous abortion risk following administration of tetanus-toxoid containing vaccines (e.g., TT, Td, TdaP). In countries where maternal and neonatal tetanus remains a public health concern, the WHO recommends that in the absence of a reliable vaccination history or completion of the childhood vaccination series, pregnant women receive tetanus vaccination [72]. Additionally, In the past half-decade, TdaP has been introduced for routine use in pregnant women in a number of countries (e.g., Argentina, Israel, New Zealand, the United Kingdom, and the United States) to protect newborn infants against pertussis [73]. Because the recommended vaccination timing for TdaP is third trimester (to optimize maternal antibody response and transfer of antibodies to the infant) [66], [73], it is anticipated that the majority of pregnant women receiving the vaccine will do so after the period of risk for a spontaneous abortion (i.e., after 22 weeks gestation). However, the existing data do not support an increased risk for spontaneous abortion following TdaP vaccination during pregnancy. One small cohort study in the United States conducted prior to routine vaccination during pregnancy reported a lower rate of spontaneous or elective abortions among 138 women receiving TdaP during pregnancy, as compared to 552 pregnant women who did not receive the vaccine (2.9% vs. 8.9%) [74]. The remaining data on spontaneous abortion risk following pertussis-containing vaccines comes from passive surveillance, including an analysis of the Vaccine Adverse Event Reporting System (VAERS) in the United States [75]. The VAERS analysis included more than 3 years of data following the recommendation to routinely vaccinate pregnant women with TdaP and found no evidence for any increase in the number of spontaneous abortion reports.

Several vaccines are not recommended for administration in pregnancy, including but not limited to those outlined below, are often inadvertently administered to women of reproductive age, and therefore unintentional exposures during pregnancy may occur. Most live vaccines are contraindicated or not recommended for use during pregnancy because of the theoretical risk of transmission of the virus to the fetus through the placenta [65].

c. Human papillomavirus vaccines:

Vaccines against human papillomavirus (HPV) are not recommended for use during pregnancy, but because they are often administered to women of reproductive age, they may be inadvertently administered during early pregnancy. Overall, the data collected as part of pregnancy registries, epidemiological studies and unintended exposures during clinical trials on HPV vaccines are mostly reassuring with respect to pregnancy outcomes data, including spontaneous abortion [76], [77], [78], [79], however specific studies of these vaccines in pregnant women were not conducted and the available safety data are insufficient to draw definite conclusions.

d. Meningococcal vaccines:

Evidence on the safety of administration of meningococcal vaccination during pregnancy is scarce. Information on spontaneous abortion risk following immunization with quadrivalent meningococcal conjugate vaccines (MCV4) is derived from passive surveillance, including a VAERS analysis that did not find any safety concerns [80]. As of June 2015, over 220 million individuals between the ages 1 and 29 years have received a new monovalent meningococcal A conjugate vaccine in 15 countries of the African belt, as part of mass immunization campaign that includes pregnant women [81], [82]. An observational cohort study conducted in Ghana did not observe any difference in risk of spontaneous abortion among 1730 immunized pregnant women (0.9%), as compared to 919 concurrent unvaccinated controls (0.7%) or 3551 historical unvaccinated controls (1.0%) [83]. To date, no data are available on the safety of monovalent meningococcal B vaccines, currently licensed for use in Europe and the United States, when administered during pregnancy.

e. Rubella and varicella vaccines:

Rubella and varicella are of specific interest because of the potential sequelae of wild-type infection in susceptible pregnant women, which could hypothetically cause congenital rubella syndrome and congenital varicella syndrome. Much of the research on safety of measles, rubella (MR) and varicella vaccines has examined congenital anomalies outcomes. Data on spontaneous abortion risk following MR vaccines are derived from adverse events registries and exposure-based registries, including a VAERS analysis of MMR (measles, mumps, rubella) vaccine and surveillance for cases during mass MR vaccination campaigns in several countries in Central and South America. Although limited, these data do not indicate any concerns related to spontaneous abortion risk [84], [85], [86], [87]. An exposure based registry of pregnant women inadvertently receiving varicella vaccine also found no evidence for a safety signal for spontaneous abortion [88].

f. Oral polio virus vaccine:

Oral polio virus vaccine, which contains live attenuated poliovirus types 1, 2, and 3, has been used to protect pregnant women and neonates against poliomyelitis since its introduction in the early 1960s. While immunization of adults with poliovirus vaccine is not routinely recommended if the series is completed during childhood, immunization of pregnant women at high risk of endemic or epidemic exposure is recommended by WHO and several national immunization technical advisory groups [65]. Limited data on spontaneous abortion following polio virus vaccination exist. In Israel, one study examined rates of spontaneous abortion during a mass oral polio virus vaccination program that was prompted by a polio epidemic in 1988. During the epidemic, over 90% of the population, including pregnant women, was administered oral polio virus vaccine, and the number of spontaneous abortions was similar both before (October through December 1987) and during the vaccination campaign (October through December 1988) [89].

*Ectopic pregnancy following immunization*

Data from clinical trials and observational studies on ectopic pregnancy following immunization are scarce.

### 1.2 Methods for the development of the case definitions and guidelines for data collection, analysis, and presentation for spontaneous abortion and ectopic pregnancy as adverse events following immunization during pregnancy

Following the process described in the overview paper [90] as well as on the Brighton Collaboration Website http://www.brightoncollaboration.org/internet/en/index/process.html, the Brighton Collaboration Abortion *Working Group* was formed in 2015 and included members of clinical, academic, public health, research and industry background. The composition of the working and reference group as well as results of the web-based survey completed by the reference group with subsequent discussions in the working group can be viewed at: http://www.brightoncollaboration.org/internet/en/index/working_groups.html.

To guide the decision-making for the case definition and guidelines, a literature search was performed using Medline, Embase and the Cochrane Libraries, including the terms abortion, miscarriage, spontaneous abortion, induced abortion, elective abortion, ectopic pregnancy, pregnancy loss, blighted ovum, anembryonic pregnancy, vaccine, immunization, maternal, pregnancy, vaccine, safety and vaccination*.* Exhaustive search strategies were implemented using appropriate key words, accepted MeSH words, and combinations thereof. All abstracts were screened for possible reports of abortion following immunization. Searches were restricted to references in English, and involving only human subjects. Multiple general medical, pediatric, obstetrics and infectious disease text books were also searched. For vaccines without published data, reviewed package inserts were reviewed (specifically for HPV9). Centers for Disease Control and Prevention Advisory Committee on Immunization Practices (CDC ACIP) presentations available on the web for relevant studies were also reviewed.

The search and screening resulted in the identification of articles with potentially relevant material for further evaluation. This literature provided several different general definitions for abortion, its epidemiology, numerous descriptions for abortion causes and/or risk factors and the diagnostic criteria put forth. Most publications addressing abortion following immunization were case reports of single cases or case series describing various pregnancy outcomes, for which terminology was very inconsistent and very few used case definitions. There was no publication identified addressing ectopic pregnancy as an outcome following immunization.

### 1.3 Rationale for selected decisions about the case definition of spontaneous abortion and the case definition of ectopic pregnancy as adverse events following immunization during pregnancy

Related term(s)

As previously mentioned, for the purposes of this document, we will be using exclusively the terms “spontaneous abortion” and “ectopic pregnancy.” There are many terms in use to describe pregnancy loss, including pregnancy failure, miscarriage, and spontaneous abortion, which can be further classified into threatened, inevitable, and missed abortion, anembryonic pregnancy, or embryonic demise.

Formulating a case definition that reflects diagnostic certainty: weighing specificity versus sensitivity

It needs to be re-emphasized that the grading of definition levels is entirely about diagnostic certainty, not clinical severity of an event. Detailed information about the severity of the event should always be recorded, as specified by the data collection guidelines.

The number of symptoms and/or signs that will be documented for each case may vary considerably. The case definition has been formulated such that the Level 1 definition is highly specific for the condition. As maximum specificity normally implies a loss of sensitivity, two additional diagnostic levels have been included in the definition, offering a stepwise increase of sensitivity from Level One down to Level Three, while retaining an acceptable level of specificity at all levels. In this way it is hoped that all possible cases of spontaneous abortion/ectopic pregnancy can be captured.

Rationale for individual criteria or decision made related to the case definition

There is a need to consider data sources and availability of existing data when defining pregnancy outcomes in research. The interpretation of data is difficult when definitions of commonly used terms differ in the literature. Flexibility and alignment with existing definitions where studies/surveillance are performed are necessary to ensure comparability and interpretation of data. Sometimes these data are not made available. As previously discussed, spontaneous abortion and ectopic pregnancy are relatively common pregnancy outcomes. Given that vaccination is also a common practice in pregnancy, there is a need for development of precise definitions of pregnancy outcomes. Careful studies are required in which appropriate controls are chosen and where the background rates of the pregnancy outcomes of concern are known. Furthermore, while recording of these common outcomes is important, it is clear that it must be done so using precise, predefined criteria in order to avoid any unmerited concern about an association of between spontaneous abortion or ectopic pregnancy and vaccination.

Determination of the gestational age at onset of the event

A proposed algorithm for estimating gestational age for studies in various community settings is presented in a related manuscript [91]. We propose utilizing this algorithm when reporting cases of spontaneous abortion/ectopic pregnancy following vaccine administration.

Gestational age cut-offs for spontaneous abortion

There is recognition that the gestational age used to define first and, in particular, second trimester spontaneous abortion varies between and even within countries (see Table 1). However, we have chosen the cut-offs presented in this document in a pragmatic manner for the purposes of classification of pregnancy outcomes. Specifically, we have chosen to define a spontaneous abortion as a pregnancy loss that occurs up to 21 weeks 6 days, with outcomes after that gestational age pertaining to the stillbirth or preterm birth categories. This then represents a “harmonized” suggested cut-off with no bearing over legal or reporting requirement issues. We strongly emphasize that this gestational age cut-off should be used for research and data collection purposes only, and is not intended to inform or impact clinical care.

**Table 1:** Conventional definitions for spontaneous abortion.

Source/Group	Gestational age (weeks)	Birth weight (grams)	Height criteria (crown-heel length)	Definition
USA (NCHS, CDC, ICD)	≤19 6/7	<350		The National Center for Health Statistics, and the Centers for Disease Control and Prevention, defines abortion as pregnancy termination prior to 20 weeks gestation or a fetus born weighing less than 500 g. Despite this, definitions vary widely according to state laws. The Model Law recommends the limit for fetal death reporting for those that occur at 350 g or more or, if the weight is unknown, of 20 completed weeks' gestation or more. A program exists for voluntary reporting of abortions of less than 20 weeks; but a fetal death certificate is not mandatory. [92]
				The International Classification of Diseases, 10th revision (ICD-10) defines spontaneous abortion as: “the loss of pregnancy from natural causes before the 20th week of pregnancy.” The definition includes the assumption that, expulsion of products of conception occurs without deliberate interference and before the fetus is viable thus weighing less than 500 g
WHO	≤21 6/7	<500	<25	The World Health Organization (WHO) defines spontaneous abortion as: “termination of pregnancy by expulsion of embryo/fetus before 22 weeks of pregnancy or below 500 g of weight. The legal requirements for the registration of fetal deaths and therefore the threshold to consider a stillbirth versus abortion vary from country to country and even within countries. WHO recommends that, if possible, all fetuses weighing at least 500 g at birth, whether alive or dead, should be included in the statistics. When information on birth weight is unavailable, the corresponding criteria for gestational age (22 completed weeks) or body length (25 cm crown-heel) should be used [93]
EMA	≤21 6/7			The European Medicines Agency uses the term spontaneous abortion as the synonym of early fetal death, which is the pregnancy ending spontaneously before 22 weeks of gestation (i.e. up to and including 21 6/7 weeks of gestation) [94]
UK (RCOG)	≤23 6/7			The United Kingdom defines abortion as a fetus born before the 24th week of pregnancy (i.e. non-viable fetus) that does not show any signs of life or a fetus expelled after the 24th week of pregnancy provided it was no longer alive by the 24th week (this fact being known or provable from the stage of development by the dead fetus) [95]

Timing post immunization in pregnancy

The time interval from immunization to onset of spontaneous abortion or ectopic pregnancy is not part of the definition, but it is recommended to be used in the data analysis to examine factors such as temporal clusters as well as determining whether the outcome of interest occurred before or after the vaccine exposure. Where feasible, details of this interval should be assessed and reported as described in the data collection guidelines (see guideline 34, Section 3.2).

### 1.4 Guidelines for data collection, analysis and presentation

As mentioned in the overview paper [90], the case definition is accompanied by guidelines that are structured according to the steps of conducting a clinical trial, i.e. data collection, analysis and presentation. Neither case definition nor guidelines are intended to guide or establish criteria for management of ill infants, children, or adults, but were instead developed to improve data comparability.

### 1.5 Periodic review

Similar to all Brighton Collaboration case definitions and guidelines, review of the definition with its guidelines is planned on a regular basis (i.e. every three to five years) or more often if needed.

## 2 Case definitions of spontaneous abortion and ectopic pregnancy^3^

For all levels of diagnostic certainty, the definitions of spontaneous abortion and ectopic pregnancy must include:

– Determination of absence of a viable pregnancy (see Table 2) AND.

**Table 2:** Current society guidelines for diagnosing spontaneous abortion.

ACOG	≤19 6/7	*First trimester*
		Non-viable, intrauterine pregnancy with either an empty gestational sac or a gestational sac containing an embryo or fetus without fetal heart activity within the first 12 6/7 weeks of gestation.
		*Findings diagnostic of spontaneous abortion (first trimester)*
		Crown-rump length (CRL) of 7 mm of greater and no heartbeat
		Mean sac diameter of 25 mm or greater and no embryo
		Absence of embryo with heartbeat 2 weeks or more after an ultrasound that showed a gestational sac without a yolk sac
		Absence of embryo with heartbeat 11 days or more after an ultrasound that showed a gestational sac with a yolk sac
		*Second trimester*
		Spontaneous pregnancy loss after the first trimester and before 20 weeks gestation
		[96]

NICE/RANZCOG/RCOG		*Evaluation with transvaginal ultrasound*
		If the mean gestational sac diameter is less than 25 mm and there is no visible fetal pole then perform a second ultrasound a minimum of 7 days after the first before making a diagnosis
		If the mean gestational sac diameter is 25 mm or more and there is no visible fetal pole then seek a second opinion on the viability of the pregnancy and/or perform a second ultrasound a minimum of 7 days after the first
		If the CRL is less than 7 mm and there is no visible heartbeat, perform a second ultrasound a minimum of 7 days after the first before making a diagnosis, and even more scans may be needed
		If the CRL is 7 mm or more and there is no visible heartbeat, then one can seek a second opinion and/or perform a second ultrasound a minimum of 7 days after the first before making a decision regarding viability of the pregnancy
		*Evaluation with transabdominal ultrasound*
		If there is no visible heartbeat when the CRL is measured then record the size of the CRL and perform a second scan a minimum of 14 days after the first scan
		Serum B-HCG should not be used alone to determine the viability of a pregnancy of unknown location.
		[5]

SGOC	≤23 6/7	*First trimester*
		Mean gestational sac diameter exceeds 8 mm without a yolk sac by transvaginal ultrasound
		Mean gestational sac diameter exceeds 16 mm without an embryo by transvaginal ultrasound
		Gestational sac greater than 20 mm without a yolk sac by transabdominal ultrasound
		Gestational sac 25 mm without an embryo by transabdominal ultrasound
		No cardiac activity in an embryo greater than 5 mm by transvaginal ultrasound or 9 mm by transabdominal ultrasound
		*Second Trimester*
		All spontaneous pregnancy losses from 13 weeks of gestation until the fetus reaches viability, 24 weeks gestation
		[4]
– Determination of fetal gestational age through maternal information OR through fetal information [91].

### 2.1 Spontaneous abortion and ectopic pregnancy ascertainment of levels of certainty

The ultimate level of certainty for the diagnosis of spontaneous abortion should incorporate the level of certainty for gestational age, such that even if the level of certainty about the diagnosis of spontaneous abortion is a Level 1, if the pregnancy dating is poor (Level 3), the diagnosis of spontaneous abortion becomes less certain, which the level should reflect, and should be reported as the same level as the pregnancy dating in this case, Level 3.

Gestational age assessment: Should be determined using the Brighton Preterm Birth Gestational Age algorithm [91].

### 2.2 First trimester spontaneous abortion

#### 2.2.1 Documentation of all aspects is required for level of ascertainment

**Level 1 (Highest level, gold standard diagnosis, maximum sensitivity and specificity)**
  Crown-rump length >7 mm and no visible heartbeat on transvaginal ultrasound
  OR
  Crown-rump length >15 mm and no visible heartbeat on transvaginal ultrasound
  OR
  Ultrasound examination demonstrating mean gestational sac diameter >25 mm and no visible embryo or yolk sac
AND
  Second transvaginal ultrasound >7 days later (or 14 days later if transabdominal) confirming diagnosis of non-viable pregnancy
  OR
  Absence of embryo with heartbeat >2 weeks after a transabdonimal scan that showed a gestational sac without a yolk sac
  OR
  Absence of embryo with heartbeat >11 days after a transvaginal scan that showed a gestational sac with a yolk sac
**OR**
  Gestational age within pre-defined range for selected abortion definition as assessed by maternal and/or fetal parameters (Level 1–2) (using the Brighton Preterm Birth Gestational Age algorithm).
AND
Positive urine or blood pregnancy test that becomes negative after 7 days
OR
Products of conception found on histopathological evaluation of pregnancy tissue
OR
Ultrasound examination demonstrating an empty uterine cavity in a woman who had clear evidence of intrauterine pregnancy on previous ultrasound examination
OR
Vaginal bleeding, external cervical or open or closed with visible expulsion of pregnancy tissue/products of conception
**Level 2 (Missing at least one confirmatory diagnostic parameter, remains sensitive and specific)**
Does not qualify as a level 1
  Crown-rump length > 7 mm and no visible heartbeat, confirmed on transvaginal ultrasound
  OR
  Crown-rump length > 15 mm and no visible heartbeat, confirmed on transvaginal ultrasound
  OR
  Mean gestational sac diameter is 25 mm or more and no visible embryo
Gestational age within pre-defined range for selected abortion definition as assessed by maternal and/or fetal parameters (Level 1–2) (using Brighton Preterm Birth Gestational Age algorithm).
**Level 3. (Less sensitive, with specificity)**
Does not qualify as a level 1 or level 2
AND
  Gestational age within pre-defined range for selected abortion definition as assessed by maternal and/or fetal parameters (Level 3) (using Brighton Preterm Birth Gestational Age algorithm).
**Level 4 (Reported spontaneous abortion with insufficient evidence to meet the case definition)**
Does not qualify as a level 1, 2 or 3
AND
  Maternal self-report or documentation in medical record without sufficient ultrasound or laboratory evidence to confirm

### 2.3 Second trimester spontaneous abortion

#### 2.3.1 Documentation of all aspects is required for level of ascertainment

The ultimate level of certainty for the diagnosis of spontaneous abortion should incorporate the level of certainty for gestational age, such that even if the level of certainty about the diagnosis of abortion is a Level 1, if the pregnancy dating is poor (Level 2), the diagnosis of abortion becomes less certain, which the level should reflect, and should be reported as the same level as the pregnancy dating in this case, Level 2.

Gestational age assessment: Should be determined using the Brighton Preterm Birth Gestational Age algorithm.

**Level 1 (Highest level, gold standard diagnosis, maximum sensitive and specificity)**
  Gestational age within pre-defined range for selected abortion definition as assessed by maternal and/or fetal-neonatal parameters (Level 1–2) (using Brighton Preterm Birth Gestational Age algorithm).
AND
  No visible heartbeat on ultrasound
  OR
  Visible expulsion of pregnancy tissues/products of conception on examination of the cervix
  OR
  Products of conception found on histopathological evaluation of uterine contents
**Level 2 (Missing at least one important parameter; remains sensitive, specific)**
Does not qualify as a level 1
AND
  Gestational age within pre-defined range for selected abortion definition as assessed by maternal and/or fetal parameters (Level 3) (using Brighton Preterm Birth Gestational Age algorithm).
AND
  No visible heartbeat on ultrasound
  OR
  Visible expulsion of pregnancy tissues/products of conception on examination of the cervix
  OR
  Products of conception found on histopathological evaluation of uterine contents
**Level 3**
  No level 3 definition for second trimester
**Level 4 (Reported abortion with insufficient evidence to meet the case definition)**
Does not qualify as a level 1 or 2
AND
  Maternal self-report or documentation in medical record without sufficient ultrasound or laboratory evidence to confirm

### 2.4 Ectopic pregnancy

#### 2.4.1 Documentation of all aspects is required for level of ascertainment

**Level 1 (Highest level, gold standard diagnosis, maximum sensitive and specificity)**
  Gestational age within pre-defined range for selected ectopic pregnancy definition as assessed by maternal and/or fetal-neonatal parameters (Level 1–2) (using Brighton Preterm Birth Gestational Age algorithm).
AND
  B-HCG serum blood test >2000 mlU/ml
AND
  TVUS showing no intrauterine pregnancy
**OR**
  Gestational age within pre-defined range for selected ectopic pregnancy definition as assessed by maternal and/or fetal-neonatal parameters (Level 1–2) (using Brighton Preterm Birth Gestational Age algorithm).
AND
  TVUS showing extrauterine pregnancy
  OR
  No products of conception found on endometrial curettage after D&C procedure
**Level 2 (Missing at least one important parameter; remains sensitive, specific)**
Does not qualify as a level 1
AND
  Gestational age within pre-defined range for selected ectopic pregnancy definition as assessed by maternal and/or fetal-neonatal parameters (Level 1–2) (using Brighton Preterm Birth Gestational Age algorithm).
AND
  TVUS showing no intrauterine pregnancy
  OR
  No products of conception found on endometrial curettage after D&C procedure
**Level 3 (Less sensitive, with specificity)**
Does not qualify as a level 1 or level 2
AND
  Gestational age within pre-defined range for selected ectopic pregnancy definition as assessed by maternal and/or fetal-neonatal parameters (Level 2–3) (using Brighton Preterm Birth Gestational Age algorithm).
AND
  Positive urine pregnancy test
AND
  No products of conception found on endometrial curettage after D&C procedure
**Level 4 (Reported ectopic pregnancy with insufficient evidence to meet the case definition)**
Does not qualify as a level 1, 2 or 3
AND
  Maternal self-report or documentation in medical record without sufficient ultrasound or laboratory evidence to confirm

## 3 Guidelines for data collection, analysis and presentation of spontaneous abortion/ectopic pregnancy

It was the consensus of the Brighton Collaboration spontaneous abortion/ectopic pregnancy *Working Group* to recommend the following guidelines to enable meaningful and standardized collection, analysis, and presentation of information about these events. However, implementation of all guidelines might not be possible in all settings. The availability of information may vary depending upon resources, geographical region, and whether the source of information is a prospective clinical trial, a post-marketing surveillance or epidemiological study, or an individual report of abortion. Also, as explained in more detail in the overview paper in this volume, these guidelines have been developed by this working group for guidance only, and are not to be considered a mandatory requirement for data collection, analysis, or presentation.

### 3.1 Data collection

These guidelines represent a desirable standard for the collection of available pregnancy outcome data following immunization to allow comparability. The guidelines are not intended to guide the primary reporting of these events to a surveillance system. Investigators developing a data collection tool based on these data collection guidelines also need to refer to the criteria in the case definition, which are not repeated in these guidelines.

Guidelines 1–46 below have been developed to address data elements for the collection of adverse event information as specified in general drug safety guidelines by the International Conference on Harmonization of Technical Requirements for Registration of Pharmaceuticals for Human Use [97], and the form for reporting of drug adverse events by the Council for International Organizations of Medical Sciences [98]. These data elements include an identifiable reporter and patient, one or more prior immunizations, and a detailed description of the adverse event, in this case, of abortion following immunization. The additional guidelines have been developed as guidance for the collection of additional information to allow for a more comprehensive understanding of spontaneous abortion/ectopic pregnancy following immunization.

#### 3.1.1 Source of information/reporter

For all cases and/or all study participants, as appropriate, the following information should be recorded:

(1) Date of report.
(2) Name and contact information of person^4^ reporting the event as specified by country specific data protection law.
(3) Relationship of the reporter to the vaccine recipient [e.g.,immunizer (clinician, nurse) attending physician, family member [indicate relationship, self [vaccine recipient], other.

#### 3.1.2 Vaccinee/control

##### 3.1.2.1 Demographics

For all cases and/or all study participants (i.e. pregnant women), as appropriate, the following information should be recorded:

(4) Case study participant identifiers (first name initial followed by last name initial) or code (or in accordance with country- specific data protection laws).
(5) Date of birth, age of patient.
(6) Gestational age.
(7) Country of residence.
(8) Occupation(s).

##### 3.1.2.2 Clinical and immunization history

For all cases and/or all study participants, as appropriate, the following information should be recorded:

(9) Past medical history, including hospitalizations, underlying diseases/disorders, pre-immunization signs and symptoms including identification of indicators for, or the absence of, a history of allergy to vaccines, vaccine components or medications; food allergy; allergic rhinitis; eczema; asthma.
(10) Any medication history (other than treatment for the event described) prior to, during, and after immunization including prescription and non-prescription medication as well as medication or treatment with long half-life or long term effect (e.g. immunoglobulins, blood transfusion and immune-suppressants) or substance abuse (e.g. narcotics or other recreational drug, alcohol or smoking).
(11) Immunization history (i.e. previous immunizations and any adverse event following immunization (AEFI), in particular occurrence of the same event after a previous immunization.
(12) Clinical confirmation of pregnancy prior to maternal immunization.

#### 3.1.3 Details of the immunization

For all cases and/or all study participants, as appropriate, the following information should be recorded:

(13) Date and time of immunization(s).
(14) Description of all vaccine (s), including name of vaccines, manufacturer, lot number, expiration date, multi or mono dose vial, volume (e.g. 0.25 Ml, 0.5 mL, etc.), dose number if part of series of immunizations against the same disease(s), description of the adjuvants and any diluents, and the manufacturer, lot number, and expiration date of any diluents used.
(15) The anatomical sites (including left or right side) of all immunizations (e.g. vaccine A in proximal left lateral thigh, vaccine B in left deltoid).
(16) Route and method of administration (e.g. intramuscular, intradermal, subcutaneous, and needle-free (including type and size), other injection devices).
(17) Needle length and gauge.
(18) If the immunization is part of:
  • Routine immunization program
  • Preventive mass immunization campaign
  • Mass immunization campaign for outbreak response
  • Domestic travel from non-endemic to endemic area
  • International travel
  • Occupational risk

#### 3.1.4 The adverse event

For all cases at any level of diagnostic certainty and for reported events with insufficient evidence, the criteria fulfilled to meet the case definition should be recorded.

Specifically document (if available):

(If data not available because of regulatory guidelines, please specify data cannot be disclosed.)

(19) Clinical description of signs and symptoms of spontaneous abortion or ectopic pregnancy, and if there was medical confirmation of the event (i.e. patient seen by physician).
(20) Date/time of onset,^5^ first observation^6^ and diagnosis^7^; as well as end of episode^8^ and final outcome.^9^
(21) Concurrent signs, symptoms, exposures and diseases.
(22) Pregnancy details:
  • Pregnancy details: date of last normal menstrual period, ultrasound examinations, antenatal care visits, pregnancy-related illnesses and complications.
  • Results of ultrasound examinations, antenatal care visits, laboratory examinations, other clinical tests, surgical and/ or pathological findings and diagnosis preferable to perform at reliable and accredited laboratories. If more than one measurement of particular parameters is taken and recorded, the value corresponding to the largest deviation from the expected normal value or range of parameter should be reported.
  • Spontaneous abortion or ectopic pregnancy details: specifically document (if available) mode of treatment (e.g. dilation and curettage, etc) and complications, if any (e.g. hemorrhage, infection, ruptured ectopic pregnancy, etc.).
(23) Measurement/testing
  • Values and units of routinely measured parameters (e.g. temperature, blood pressure) – in particular those indicating the severity of the event;
  • Method of measurement (e.g. type of thermometer, oral or other route duration of measurement, etc.);
  • Results of laboratory examinations, surgical and/or pathological findings and diagnoses if present.
(24) Treatment given for the event, especially specify what and dosing, if applicable.
(25) Outcome 8 at last observation (e.g. for an event that meets the case definition of spontaneous abortion, it results in death of the embryo/fetus but not necessarily the mother). Add descriptions if maternal death occurred. Also, for multiple gestation, if concomitant twin death occurred. For example:
  • Recovery to pre- immunization health status
  • Spontaneous resolution
  • Ongoing treatment/recovering
  • Persistence of the event/unresolved
  • Significant complications of treatment/sequelae
  • Maternal death and description of any other outcome
(26) Objective clinical evidence supporting classification of the event as “serious”^10^ (i.e. results in death of the embryo/fetus, hospitalisation of the mother).
(27) Exposures other than the immunization before and after immunization (e.g. trauma, induced, environmental) considered potentially relevant to the reported event.

#### 3.1.5 Miscellaneous/general

(28) The duration of follow-up reported during the surveillance period should be predefined likewise. It should aim to continue to resolution of the event (i.e. the outcome of the pregnancy is captured).
(29) Methods of data collection should be consistent within and between study groups, if applicable.
(30) Follow-up of cases should attempt to verify and complete the information collected as outlined in data collection guidelines 1–27.
(31) Guidance should be provided to optimize the quality and completeness of information.
(32) Reports of pregnancy outcomes should be collected throughout the study period regardless of the time elapsed between immunization and the adverse event. If this is not feasible due to the study design, the study periods during which safety data are being collected should be clearly defined.
(33) The safety monitoring should take into account:
  • Biologic characteristics of the vaccines (e.g., live attenuated versus inactivated component vaccines).
  • The vaccine preventable-disease.
  • Non clinical and clinical data obtained previously and
  • Characteristics of the target population (e.g., nutrition, underlying disease like immunocompromised illness).
(34) Methods of data collection should be consistent within and between study groups or surveillance systems, if applicable.
(35) Reports of pregnancy outcomes should be collected throughout the study period regardless of the time elapsed between immunization and the adverse event. If this is not feasible due to the study design, the study periods during which safety data are being collected should be clearly defined.

### 3.2 Data analysis

The following guidelines represent a desirable standard for analysis of data on spontaneous abortion and ectopic pregnancy to allow for comparability of data, and are recommended as an addition to data analyzed for the specific study question and setting.

(36) Reported events should be classified in one of the following five categories including the three levels of diagnostic certainty. Events that meet the case definition should be classified according to the levels of diagnostic certainty as specified in the case definition. Events that do not meet the case definition should be classified in the additional categories for analysis.

**Event classification in 5 categories**^11^

**Event meets case definition**

(1) Level 1: Criteria as specified in the case definition
(2) Level 2: Criteria as specified in the case definition
(3) Level 3: Criteria as specified in the case definition

**Event does not meet case definition**

***Additional categories for analysis***

(4) Reported event with insufficient evidence to meet the case definition^12^
(5) Not a case of spontaneous abortion/ectopic pregnancy^13^
(37) The interval between immunization and reported abortion could be defined as the date/time of immunization (last vaccination) to the date/time of onset 4 of the event, consistent with the definition. It is important to note that timing of fetal demise may differ by days to weeks from the time when a spontaneous abortion or ectopic pregnancy is clinically recognized. If few cases are reported, the concrete time course could be analyzed for each; for a large number of cases, data can be analyzed in the following increments for identification of temporal clusters:

**Subjects with spontaneous abortion or ectopic pregnancy by interval to presentation**

**Table:** 

Interval^∗^	Number
<24 h after immunization	
2–<7 days after immunization	
8–<42 days after immunization	
>42 days after immunization	
Weekly unit increments thereafter	
**Total**

(38) If more than one measurement of a particular criterion is taken and recorded, the value corresponding to the greatest magnitude of the adverse experience could be used as the basis for analysis. Analysis may also include other characteristics like qualitative patterns of criteria defining the event.
(39) The distribution of data (as numerator and denominator data) could be analyzed in predefined increments (e.g. measured values, times), where applicable. Increments specified above should be used. When only a small number of cases is presented, the respective values or time course can be presented individually.
(40) Data on spontaneous abortion/ectopic pregnancy obtained from subjects receiving a vaccine should be compared with those obtained from an appropriately selected and documented control group(s) to assess background rates in non-exposed populations, and should be analyzed by study arm and dose where possible, e.g. in prospective clinical trials. It should be emphasized that because risk of spontaneous abortion/ectopic pregnancy is time-dependent (i.e. inversely related to week of gestation [42], choosing appropriate control groups is paramount. For example, if a group receiving vaccination is compared to a group receiving a placebo but the women in the vaccine arm receive the vaccine at 6 weeks gestation and the control women receive the placebo at 8 weeks, the rate of SA after the vaccination will be higher, reflecting differences in background risk.

### 3.3 Data presentation

These guidelines represent a desirable standard for the presentation and publication of data on abortion following immunization to allow for comparability of data, and are recommended as an addition to data presented for the specific study question and setting. Additionally, it is recommended to refer to existing general guidelines for the presentation and publication of randomized controlled trials, systematic reviews, and meta-analyses of observational studies in epidemiology (e.g. statements of Consolidated Standards of Reporting Trials (CONSORT) [99], of Improving the quality of reports of meta-analyses of randomized controlled trials (QUORUM) [100], and of Meta-analysis Of Observational Studies in Epidemiology (MOOSE) [101], respectively).

(41) All reported events should be presented according to the categories listed in guideline 36.
(42) Data on possible abortion events should be presented in accordance with data collection guidelines 1–35 and data analysis guidelines 36–40.
(43) Data should be presented with numerator and denominator (n/N) (and not only in percentages), if available.
  Although immunization safety surveillance systems denominator data are usually not readily available, attempts should be made to identify approximate denominators. The source of the denominator data should be reported and calculations of estimates be described (e.g. manufacturer data like total doses distributed, reporting through Ministry of Health, coverage/population based data, etc.).
(44) The incidence of cases in the study population should be presented and clearly identified as such in the text.
(45) If the distribution of data is skewed, median and inter-quartile range are usually the more appropriate statistical descriptors than a mean. However, the mean and standard deviation should also be provided.
(46) Any publication of data on pregnancy outcomes should include a detailed description of the methods used for data collection and analysis as possible. It is essential to specify:
  • The study design;
  • The method, frequency and duration of monitoring for pregnancy outcomes;
  • The trial profile, indicating participant flow during a study including drop-outs and withdrawals to indicate the size and nature of the respective groups under investigation;
  • The type of surveillance (e.g. passive or active surveillance);
  • The characteristics of the surveillance system (e.g. population served, mode of report solicitation);
  • The search strategy in surveillance databases;
  • Comparison group(s), if used for analysis;
  • The instrument of data collection (e.g. standardized questionnaire, diary card, report form);
  • Whether the day of immunization was considered “day one” or “day zero” in the analysis;
  • Whether the date of onset 4) and/or the date of first observation 5 and/or the date of diagnosis 6 was used for analysis; and
  • Use of this case definition, in the abstract or methods section of a publication^14^.

## Disclaimer

The findings, opinions and assertions contained in this consensus document are those of the individual scientific professional members of the working group. They do not necessarily represent the official positions of each participant’s organization. Specifically, the findings and conclusions in this paper are those of the authors and do not necessarily represent the views of their respective institutions.

## Footnotes

3 The case definition should be applied when there is no clear alternative diagnosis for the reported event to account for the combination of symptoms.

4 If the reporting center is different from the vaccinating center, appropriate and timely communication of the adverse event should occur.

5 The date and/or time of onset is defined as the time post immunization, when the first sign or symptom indicative for abortion occurred. This may only be possible to determine in retrospect.

6 The date and/or time of first observation of the first sign or symptom indicative for abortion can be used if date/time of onset is not known.

7 The date of diagnosis of an episode is the day post immunization when the event met the case definition at any level.

8 The end of an episode is defined as the time the event no longer meets the case definition at the lowest level of the definition.

9 Example: recovery to pre-immunization health status, spontaneous resolution, therapeutic intervention, persistence of the event, sequelae, death.

10 An AEFI is defined as serious by international standards if it meets one or more of the following criteria: 1) it results in death, 2) is life-threatening, 3) it requires inpatient hospitalisation or results in prolongation of existing hospitalisation, 4) results in persistent or significant disability/incapacity, 5) is a congenital anomaly/birth defect, 6) is a medically important event or reaction. For abortion, the event meets the definition of serious (i.e. it results in death of the embryo/fetus).

11 To determine the appropriate category, the user should first establish, whether a reported event meets the criteria for the lowest applicable level of diagnostic certainty, e.g. Level three. If the lowest applicable level of diagnostic certainty of the definition is met, and there is evidence that the criteria of the next higher level of diagnostic certainty are met, the event should be classified in the next category. This approach should be continued until the highest level of diagnostic certainty for a given event could be determined. major criteria can be used to satisfy the requirement of minor criteria. If the lowest level of the case definition is not met, it should be ruled out that any of the higher levels of diagnostic certainty are met and the event should be classified in additional categories four or five.

12 If the evidence available for an event is insufficient because information is missing, such an event should be categorised as “reported abortion with insufficient evidence to meet the case definition”.

13 An event does not meet the case definition if investigation reveals a negative finding of a necessary criterion (necessary condition) for diagnosis. Such an event should be rejected and classified as “Not a case of abortion”.

14 Use of this document should preferably be referenced by referring to the respective link on the Brighton Collaboration website (http://www.brightoncollaboration.org).
